# Recent Treatment Options for Primary Colonic Leiomyosarcoma: A Case Report and a Review of the Literature

**DOI:** 10.70352/scrj.cr.25-0532

**Published:** 2025-10-25

**Authors:** Akira Dejima, Masashi Momiyama, Kentaro Nakajima, Mao Tsuru, Jun Sakuma, Atsuki Nagao, Hirotsugu Hashimoto, Keita Uchino, Kazushige Kawai, Shouichi Satou

**Affiliations:** 1 Department of Colorectal Surgery, Tokyo Metropolitan Cancer and Infectious Diseases Center, Komagome Hospital, Tokyo, Japan; 2 Department of Surgery, NTT Medical Center Tokyo, Tokyo, Japan; 3 Department of Diagnostic Pathology, NTT Medical Center Tokyo, Tokyo, Japan; 4 Department of Medical Oncology, NTT Medical Center Tokyo, Tokyo, Japan

**Keywords:** primary colonic leiomyosarcoma, laparoscopic resection, adjuvant chemotherapy

## Abstract

**INTRODUCTION:**

Primary colonic leiomyosarcoma (LMS) accounts for only 0.12% of colonic malignancies. However, it is highly aggressive and associated with a poor prognosis. While radical surgical resection is considered the mainstay of treatment, the optimal surgical approach and the role of adjuvant chemotherapy remain unclear due to the small number of reported cases.

**CASE PRESENTATION:**

A 45-year-old, male patient presented with hematochezia. Colonoscopy revealed a 40-mm, type 1 tumor in the sigmoid colon. Immunohistochemical analysis of a biopsy specimen found atypical spindle cell proliferation, positivity for desmin and h-caldesmon, and negativity for CK AE1/AE3, indicating smooth muscle differentiation. Contrast-enhanced CT demonstrated a 40-mm, enhancing mass without a lymph node or distant metastasis. The patient underwent a laparoscopic sigmoidectomy with lymph node dissection. Pathological examination confirmed a 40 × 42 × 35 mm, French Federation of Cancer Centers (FNCLCC) grade 2 leiomyosarcoma with partial serosal exposure, negative margins, and no nodal involvement (0/42). Immunohistochemistry revealed positivity for α-SMA, desmin, h-caldesmon, and calponin, with a Ki-67 index of 60%–80%. Postoperatively, the patient received six cycles of adjuvant chemotherapy with doxorubicin and ifosfamide. A follow-up examination at 3 years and 6 months found no recurrence.

**CONCLUSIONS:**

This report underscores the potential efficacy of minimally invasive surgery and adjuvant chemotherapy in some patients with primary colonic LMS, which in the present case was successfully managed with laparoscopic resection followed by adjuvant chemotherapy. More evidence is needed to establish a standard treatment strategy.

## Abbreviations

GIST
: gastrointestinal stromal tumor
LMS
: leiomyosarcoma

## INTRODUCTION

Since the recognition of GIST as a distinct entity, many tumors previously described as gastrointestinal LMS have been reclassified as GIST. Genuine gastrointestinal LMS is now known to be exceedingly rare. Primary colonic LMS accounts for only 0.12% of colonic malignancies, is highly aggressive, and has a poor prognosis.^1,2)^ Surgical resection is the only treatment, but due to its rarity, there are few reports, and no standard surgery (open or laparoscopic) or postoperative adjuvant chemotherapy has been established.

Herein, we report a case of primary LMS of the sigmoid colon which was resected laparoscopically, then treated with postoperative adjuvant chemotherapy consisting of doxorubicin and ifosfamide.

## CASE PRESENTATION

**Fig. 1:**
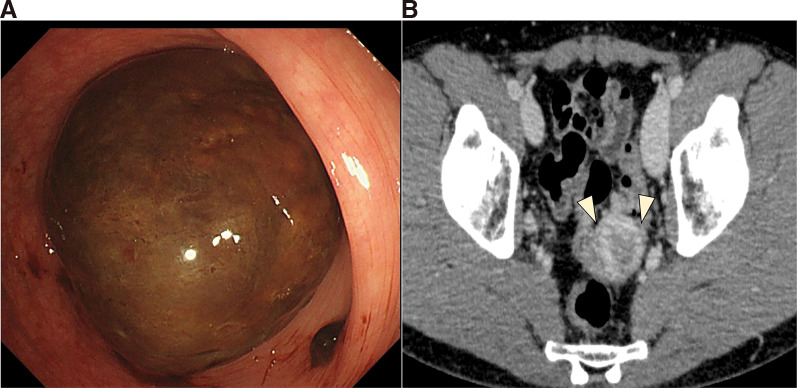
Preoperative findings. (**A**) Lower gastrointestinal endoscopy findings. A type 1 tumor, measuring 40 mm was observed in the sigmoid colon. (**B**) Contrast-enhanced CT findings. A tumor with enhancement, measuring 40 mm, was observed in the sigmoid colon, but there was no clear evidence of a lymph node or distant metastasis.

The patient was a 45-year-old male. Lower gastrointestinal endoscopy performed for hematochezia revealed a black, type 1 tumor, measuring 40 mm in the sigmoid colon at a distance of 30 cm from the anal verge (**Fig. 1A**). **Figure 1A** shows the tumor prior to biopsy. Histological analysis of a biopsy specimen found a proliferation of atypical spindle cells. Immunohistochemical analysis demonstrated positivity for desmin and h-caldesmon and negativity for CK AE1/AE3. Scattered, weakly p53-positive cells indicating the *TP53* wild-type pattern were found. These findings indicated that the lesion was a malignant, mesenchymal tumor with smooth muscle differentiation, for which the differential diagnosis included leiomyosarcoma and sarcomatous element of carcinosarcoma. Blood test findings demonstrated normal tumor marker values, with CEA at 0.6 ng/mL and CA19-9 at 6.0 U/mL. Contrast-enhanced CT identified a 40-mm tumor with enhancement in the sigmoid colon but no evidence of a lymph node or distant metastasis (**Fig. 1B**).

A multidisciplinary conference concluded that there was no multi-organ invasion and that safe, complete laparoscopic resection was feasible. Following the principles of curative surgery for colon cancer, a laparoscopic sigmoidectomy with lymph node dissection was performed.

**Fig. 2:**
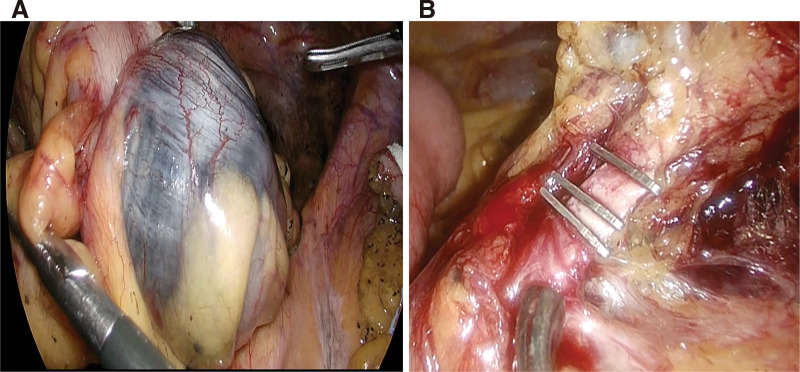
Intraoperative findings. (**A**) The tumor and tattoo markings were observed on the proximal side of the sigmoid colon. (**B**) The inferior mesenteric artery was ligated at its root.

Laparoscopic surgery was performed with 5 ports. The tumor and tattoo markings were identified on the proximal side of the sigmoid colon (**Fig. 2A**). Mobilization of the sigmoid colon was conducted from the medial side, and the inferior mesenteric artery was ligated at its root (**Fig. 2B**). After securing a 10-cm margin around the tumor, the sigmoid colon was resected. An end-to-end anastomosis was performed using the double-stapling technique. The operative time was 2 hours and 42 minutes, and the estimated blood loss was 5 mL.

**Fig. 3:**
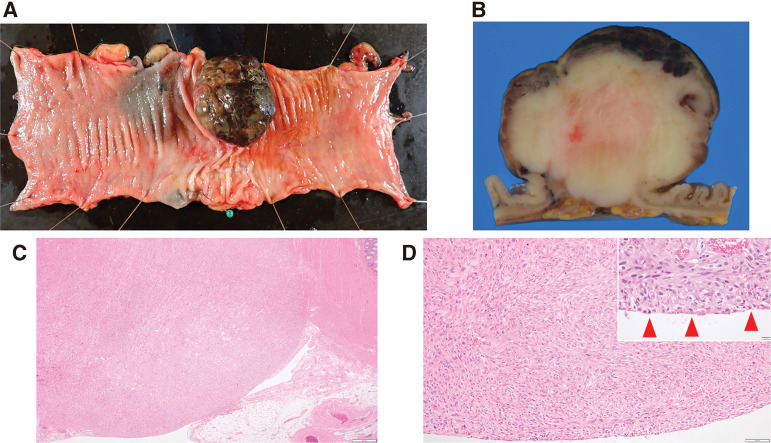
Resection sample and histological examination. (**A**) The lesion was a type 1 tumor measuring 40 × 42 × 35 mm. (**B**) The cut surface was solid and grayish white with partial exposure to the serosal surface. (**C**) Proliferation of atypical spindle-shaped cells corresponding to the macroscopic lesion was observed. (**D**) Exposure to the serosal surface was confirmed (arrowheads).

Analysis of a resection specimen revealed a type 1 tumor measuring 40 × 42 × 35 mm (**Fig. 3A**). On the cut surface, the tumor appeared solid and grayish white, with partial exposure to the serosal surface. The mucosal surface appeared dark brown to black with necrosis, in line with the endoscopic findings (**Fig. 3B**). Histopathological analysis found proliferation of atypical, spindle-shaped cells corresponding to the macroscopic lesion (**Fig. 3C**), thus confirming the exposure to the serosal surface (**Fig. 3D**). There were 14 mitoses per 10 high power fields. No significant tumor necrosis was observed.

**Fig. 4:**
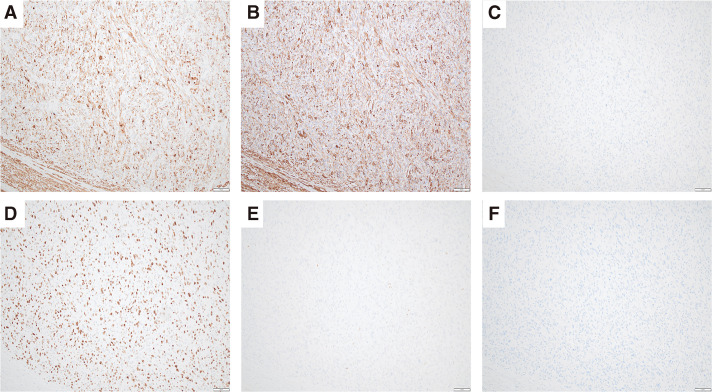
Immunohistochemistry. (**A**) Desmin positive. (**B**) h-caldesmon positive. (**C**) c-kit negative. (**D**) The Ki-67 labeling index was 60%–80%. (**E**) p53 showing scattered, weakly positive cells (wild-type pattern). (**F**) MDM2 negative.

Immunohistochemical analysis found the atypical, spindle-shaped cells to be positive for α-SMA, desmin, h-caldesmon, and calponin but negative for estrogen receptor, c-kit, DOG-1, MDM2, CDK4, and CK AE1/AE3 (**Fig. 4**). p53 staining found scattered, weakly positive cells indicating a wild-type pattern. The Ki-67 labeling index was 60%–80%.

These findings led to the diagnosis of primary leiomyosarcoma of the sigmoid colon. The tumor was grade 2 on the French Federation of Cancer Centers (FNCLCC) grading system. All surgical margins were negative, and 42 lymph nodes were harvested, none of which contained a metastasis. The postoperative course was uneventful, and the patient was discharged on POD 7.

Six cycles of adjuvant chemotherapy consisting of doxorubicin and ifosfamide were administered. Each cycle consisted of doxorubicin 30 mg/m^2^ on days 1 and 2 and ifosfamide 2.0 g/m^2^ on days 1–3 and was repeated every 3 weeks.

Postoperative follow-up consisted of a clinical evaluation and blood tests every 3 months, contrast-enhanced CT every 4 months, and a colonoscopy every 6 months for the first 2 years, followed by an annual colonoscopy thereafter. At postoperative 3 years and 6 months, the patient has experienced no recurrence and is doing well.

## DISCUSSION

Most non-epithelial tumors or mesenchymal tumors of the gastrointestinal tract were once thought to be leiomyomas or LMSs. However, after Hirota et al. established the concept of GIST, many tumors previously considered LMS have now been reclassified as GISTs.^3)^ Miettinen et al.’s re-evaluation of the immunohistochemistry of past cases of gastrointestinal mesenchymal tumor found that 92.4% were GISTs while only 5.6% were LMSs.^4)^ Both GISTs and LMSs are most common in individuals aged 50–60 years, with GIST showing no gender preference and LMS being slightly more prevalent in male individuals.^5)^ Immunohistochemical examination is essential for diagnosing LMS, for which confirmation of negativity for the GIST markers, c-kit and CD34, and positivity for smooth muscle cell markers, such as α-SMA, desmin, and h-caldesmon are essential. The presence of mitoses and nuclear atypia are also important in diagnosing LMS.^6,7)^

Accurately differentiating between GIST and LMS is crucial because of the different treatment strategies involved. GIST can be treated effectively with tyrosine kinase inhibitors, whereas LMS, which lacks kit mutations, does not respond well to these inhibitors.^7)^ Other systemic therapies have limited efficacy, leaving surgery as the standard treatment.

The 5-year overall survival rate for primary colonic LMS is 45.6%, and the mean overall survival time is 95.5 ± 18.6 months.^8)^ Although prognostic factors are not well known, the patient’s age, tumor differentiation, and tumor size have been identified as potential predictors of disease-specific survival and distant recurrence.^9)^ Despite this, colonic LMS demonstrates a high risk of recurrence even after complete resection; it recurs most often in distant rather than local sites, has a median time to recurrence of 13–18 months, and in about 60% of cases recurs within the first 2 years.^10)^ However, late recurrences beyond 5 years after resection have also been reported, underscoring the need for long-term surveillance. The lung is the most frequent site of metastasis (64%–75% of cases), followed by the liver and peritoneum, with the latter often being associated with local recurrences. Less common, metastatic sites include bone and soft tissue while the brain, skin, and thyroid involvement are rare.^11)^ As of this writing, our patient has remained recurrence-free for 3 years and 6 months under regular follow-up with CT, blood tests, and colonoscopy, but continued, long-term monitoring is required to assess the prognosis.

The standard treatment for primary colonic LMS is complete tumor excision. While open surgery is the conventional approach, the current era of minimally invasive surgery has sparked ongoing discussions about the optimal, surgical method. Six cases of laparoscopic surgery for primary colonic LMS, including the present case, have been reported in PubMed (**Table 1**). Yahagi et al.,^12)^ who first performed laparoscopic surgery for this condition, suggested that this surgical technique should be considered only for primary colonic LMS smaller than 5 cm owing to the poor prognosis of larger tumors. However, Pagliai et al. and Bananzadeh et al. have reported achieving complete, laparoscopic resection of primary colonic LMS larger than 5 cm.^13,14)^ Although the present case involved a primary colonic LMS smaller than 5 cm, the widespread adoption of laparoscopic surgery today suggests that the laparoscopic approach may be feasible regardless of tumor size as long as the treatment facility has an established, laparoscopic technique and there are no issues with tumor manipulation or field of view.

**Table 1:** Laparoscopic surgery for primary colonic LMS in PubMed

No.	Author	Year	Age	Sex	Location	Tumor size (mm)	Surgery	Postoperative adjuvant chemotherapy	Postoperative observation period	Prognosis
1	Yahagi^12)^	2019	46	M	S	28	Lap-S	–	12 months	No recurrence Alive
2	Bananzadeh^14)^	2021	48	M	S	80	Lap-S	–	ND	No recurrence Alive
3	Bananzadeh^14)^	2021	49	M	D	40	Lap-LHR	ND	ND	Recurrence Alive
4	Wong^21)^	2021	59	M	A	20	Lap-RHC	–	6 months	No recurrence Alive
5	Pagliai^13)^	2022	51	M	S	60	Lap-S	–	12 months	No recurrence Alive
6	Our case		45	M	S	42	Lap-S	+	42 months	No recurrence Alive

Lap-LHC, laparoscopic left hemicolectomy; Lap-RHC, laparoscopic right hemicolectomy; Lap-S, laparoscopic sigmoid colectomy; ND, no data

Lymph node metastases of LMS are rare, and distant metastases are usually hematogenous. Large-scale studies of soft tissue sarcomas have found an incidence of only about 1.3%, and prophylactic lymph node dissection has proven to be ineffective in improving the prognosis. For these reasons, routine dissection is generally not recommended. However, a recent SEER database analysis reported lymph node metastasis in 7.9% of gastrointestinal LMS cases.^15)^ In the present case, therefore, en bloc resection with D3 lymph node dissection was performed to ensure complete tumor removal.

As of yet, there is no consensus on the optimal adjuvant therapy regimen for primary colonic LMS. Because LMS is generally resistant to chemotherapy and radiotherapy, some studies have suggested that postoperative adjuvant therapy is unnecessary after a complete resection. However, for patients with a high risk of recurrence, multi-agent regimens, such as doxorubicin plus dacarbazine or doxorubicin plus ifosfamide, have provided some clinical benefit.^16)^ In the present case, although R0 resection was achieved with sufficient margins, adjuvant chemotherapy with doxorubicin and ifosfamide was administered due to the increased risk of recurrence associated with FNCLCC grade 2 and partial serosal exposure.^17)^ This regimen, which is among the most widely used for soft tissue sarcomas, has a response rate of 20%–30% in cases of advanced disease.^18)^ The current guidelines for the treatment of soft tissue sarcoma^19)^ also recommend the use of adjuvant chemotherapy, citing meta-analyses and randomized trials demonstrating the survival benefit of doxorubicin-based regimens. Furthermore, a pooled analysis of 2 randomized trials demonstrated improved overall survival, particularly in patients with an R1 resection.^20)^ On this basis and considering the patient’s relatively young age and good performance status, doxorubicin plus ifosfamide was chosen to reduce the recurrence risk.

## CONCLUSIONS

We reported a case of primary colonic LMS which was resected laparoscopically and treated with postoperative adjuvant chemotherapy. Given the rarity of primary colonic LMS, more data are needed to establish a standard treatment protocol.

## DECLARATIONS

### Availability of data and materials

All the data generated or analyzed during this study are included in this article.
